# EYA4 serves as a prognostic biomarker in hepatocellular carcinoma and suppresses tumour angiogenesis and metastasis

**DOI:** 10.1111/jcmm.14309

**Published:** 2019-04-07

**Authors:** Fangming Gu, Shengxian Yuan, Lei Liu, Peng Zhu, Yuan Yang, Zeya Pan, Weiping Zhou

**Affiliations:** ^1^ The Third Department of Hepatic Surgery Shanghai Eastern Hepatobiliary Surgery Hospital Shanghai China

**Keywords:** angiogenesis, c‐JUN, EYA4, hepatocellular carcinoma, metastasis

## Abstract

Eye absent homolog 4 (EYA4) has been demonstrated to be down‐regulated in hepatocellular carcinoma (HCC), but its biological function and the mechanism in HCC angiogenesis and metastasis remain largely unknown. Herein, we showed that EYA4 expression was frequently low in HCC tissue samples compared with matched adjacent non‐tumourous tissues. In the analysis of 302 HCC specimens, we revealed that decreased expression of EYA4 correlated with tumour differentiation status. Univariate and multivariate analyses identified EYA4 as an independent risk factor for recurrence‐free survival (RFS) and overall survival (OS) among the 302 patients. Functional assays showed that forced expression of EYA4 suppressed HCC cell migration, invasion and capillary tube formation of endothelial cells in vitro, as well as in vivo tumour angiogenesis and metastasis in a mouse model. Furthermore, mechanism study exhibited that EYA4 could inhibit HCC angiogenesis and metastasis by inhibiting c‐JUN/VEGFA pathway. Together, we provide proof that EYA4 is a novel tumour suppressor in HCC and a new prognostic biomarker and therapeutic target in HCC.

## 1 INTRODUCTION

Hepatocellular carcinoma (HCC) is the fifth most fatal cancer and the third most common cause of cancer‐related deaths in the world, and the number of newly diagnosed cases continues to grow.^1^ Prognosis among patients with HCC remains poor after chemotherapy or pharmacotherapy on account of high rates of recurrence and metastasis.^2^ Hence, it is imperative to further investigate the molecular mechanisms underlying the progression and recurrence of HCC and to ascertain potential therapeutic targets to improve HCC treatment.

The Eyes Absent (EYA) family of proteins represents members of a highly conserved regulatory network dealing with cell fate determination of organisms from insects to humans. The EYA family contains four members—*EYA1*, *EYA2*, *EYA3* and *EYA4*—which are the novel vertebrate genes related to the eyes absent gene in *Drosophila*.^3^ EYA proteins are dual‐role phosphatases whose threonine phosphatase and tyrosine phosphatase activities are carried out by different domains.^4^ It is generally recognized that EYAs work as transcriptional coactivators recruited by the SIX protein,^5^, ^6^ and transactivation of the SIX–EYA complex depends on the tyrosine phosphatase activity of EYA.^7^ As mentioned above, EYA4 belongs to the EYA family and is reported to be dysregulated in some human cancers.^8^ EYA4 is inactivated in non–small cell lung cancer, and blocked *EYA4* expression is associated with poor prognosis in sporadic lung cancers.^9^ In addition, *EYA4* is a promising tumour suppressor gene because it controls the up‐regulation of DKK1 and blocks the Wnt signalling pathway in colorectal cancer.^10^ Likewise, lower levels of EYA4 expression are observed in HCC tissues and are an independent predictor of both shorter disease‐free survival (DFS) and overall survival (OS).^11^ And more recently, EYA4 was shown to inhibit HCC cell growth and invasion by suppressing NF‑κB‑dependent RAP1 transactivation.^12^ Nevertheless, the precise role that EYA4 plays in HCC angiogenesis and metastasis remain largely unknown.

Herein, we report that low expression of EYA4 is closely related to tumour differentiation status and poor prognosis of HCC. Our experiments showed that forced expression of EYA4 suppressed HCC angiogenesis and metastasis via inhibiting c‐JUN/VEGFA pathway. These results provide not only a clearer understanding of the involvement of EYA4 in HCC progression but also a potential therapeutic target in HCC.

## 2 MATERIALS AND METHODS

### 2.1 Patients and clinical tissue samples

Fresh HCC tissue samples and matched normal liver tissue samples were obtained from 10 patients with HCC during hepatic surgery at the Shanghai Eastern Hepatobiliary Surgery Hospital (Shanghai, China). The samples were immediately frozen in liquid nitrogen for the subsequent experiments. Another 302 paired tissues used for tissue microarray (TMA) analysis were randomly collected from patients who underwent resection of hepatic haemangiomas in this hospital. All the patients provided written informed consent, and the study protocol was approved by the Ethics Committee of the Shanghai Eastern Hepatobiliary Surgery Hospital.

### 2.2 Immunohistochemical staining

This staining was performed as described previously.^13^ After fixing with formalin and embedding in paraffin, tissue sections were prepared, deparaffinized and rehydrated and then subjected to antigen retrieval with citrate buffer (pH 6.0). The tissue slices were incubated in 0.3% H_2_O_2_ and blocked with 1% BSA for another 30 min. Next, the slices were incubated with primary antibodies at 4°C for 12 h. After that, the slices were probed with a secondary antibody at room temperature for 30 min. The Dako ChemMate^TM^ Envision^TM^ Detetcion Kit (DaKo, Glostrup, Denmark) was subsequently applied to detect the primary antibodies. The tissue slides were lightly counterstained with haematoxylin and photographed using an Olympus microscope (Model BX40F4, Tokyo, Japan). Integrated optical density (IOD) was measured by means of Image‐Pro Plus 6.0 (IPP) and EYA4 down‐regulation was defined as IOD weaker in HCC than in a paired non‐tumourous tissue sample.

### 2.3 Cell lines and cell culture

Human HCC cell lines, SMMC‐7721 and HCC‐LM3, were obtained from the Cell Bank of Type Culture Collection of Chinese Academy of Sciences (Shanghai, China). All the cell lines were cultured in the DMEM medium (Gibco BRL, Rockville, MD) supplemented with 10% of fetal bovine serum (Sigma, St. Louis, MO), 100 U/mL penicillin and 100 μg/mL streptomycin (Invitrogen, Carlsbad, CA). Each cell line was cultivated at 37°C and 5% CO_2_ in a humidified incubator.

### 2.4 Lentiviral transduction

The lentivirus overexpressing EYA4 was purchased from Hanbio (Shanghai, China). The corresponding empty lentiviral vector served as a negative control. The EYA4 knockdown lentivirus was also obtained from Hanbio. SMMC‐7721 and HCC‐LM3 cells were transduced with the recombinant lentivirus in the presence of 10 μg/mL Polybrene. Forty‐eight hours later, Western blot analysis was carried out for determining knockdown efficiency.

### 2.5 Western blot analysis

This analysis was conducted as previously described.^14^ Cells were lysed in RIPA buffer to obtain total protein samples and the proteins were separated by SDS‐PAGE in a 10% gel and then electrophoretically transferred to a nitrocellulose membrane (Bio‐Rad, Hercules, CA). The membranes were blocked with 5% non‐fat milk and then incubated with the following primary antibodies: anti‐EYA4, anti‐c‐JUN, anti‐p‐c‐JUN(ser73), anti‐VEGFA and anti‐CD31 (Cell Signalling Technology, Beverly, MA) overnight at 4°C. GAPDH served as a loading control. The membranes were incubated with a horseradish peroxidase–conjugated secondary antibody. The protein bands were detected with the ECL Plus Developing System (Amersham Biosciences; Piscataway, NJ).

### 2.6 Wound‐healing assay

This assay was performed to assess the cell migration ability. SMMC‐7721 and HCC‐LM3 cells with or without ectopic expression of EYA4 seeded in 6‐well plates were cultured to 80%–90% confluence and then pretreated with 10 μg/mL mitomycin C (Sigma) for 2 h. The cell monolayer was scratched with a sterile 200 μL pipette tip, and the detached cells were removed with a PBS wash. After cultivation in the complete DMEM medium for 48 h, the cells were photographed. The distance traveled by the cells between the two boundaries of the wound was calculated.

### 2.7 Transwell migration and invasion assays

SMMC‐7721 and HCC‐LM3 cells with or without ectopic expression of EYA4 were seeded onto the membrane of the upper chamber with Matrigel in a serum‐free medium. The lower chamber was filled with the complete DMEM medium (containing 10% of fetal bovine serum as a chemoattractant). After incubation at 37°C for 12 h, the invading cells that got attached to the lower surface of the membrane were stained with crystal violet and counted under a microscope. The migration ability of HUVEC cells was tested using a BD Transwell assay insert with a non‐coated membrane.

### 2.8 Tube formation assay

HUVECs (1 × 10^4^ cells/well) were pre‐cultured in medium without serum for 6 h and seeded over the Matrigel‐coated 96‐well plate. After attached, the cells were cultured in the indicated conditioned media of SMMC‐7721 and HCC‐LM3 cell supernatants for 6 h. The total tube area was quantified as mean relative tube length obtained from image analysis of five random microscopic fields using Image J software.

### 2.9 In vivo assays

The animal experiment was approved by the Scientific Investigation Board of the Second Military Medical University (Shanghai, China). Four‐week‐old female BALB/c nude mice were used in this experiment. HCC‐LM3 cells (2 × 10^6^) that were transfected with the EYA4‐overexpressing lentivirus and the corresponding control cells were injected subcutaneously into the flanks of nude mice (five per group). Four weeks later, all the mice were euthanized and the tumours were excised. After fixing with 10% neutral buffered formalin, these tumours were embedded in paraffin, sectioned and then subjected to immunohistochemical (IHC) staining with antibodies against EYA4, c‐JUN, VEGFA and CD31. For lung metastasis assay, 2 × 10^6^ HCC‐LM3 cells overexpressing EYA4 or vector were injected into nude mice through the tail vein (five per group). After 8 weeks, the mice were sacrificed and their lungs were harvested, fixed and prepared for standard histological examination.

### 2.10 Statistical analysis

Data were expressed as mean ± standard deviation. Statistical analyses were conducted on a personal computer in the SPSS 23.0 statistical software (SPSS Inc, Chicago, IL). Differences between two groups were assessed by unpaired Student's *t* test and qualitative variables were analysed by the χ^2^ test. Kaplan‐Meier analyses with the log rank test were performed to evaluate recurrence‐free survival (RFS) and OS. A Cox proportional hazards model was employed to analyse the independent factors affecting RFS and OS. Data with *P* < 0.05 were considered significant.

## 3 RESULTS

### 3.1 EYA4 is repressed in HCC and serves as a prognostic factor in patients with HCC

**Figure 1:**
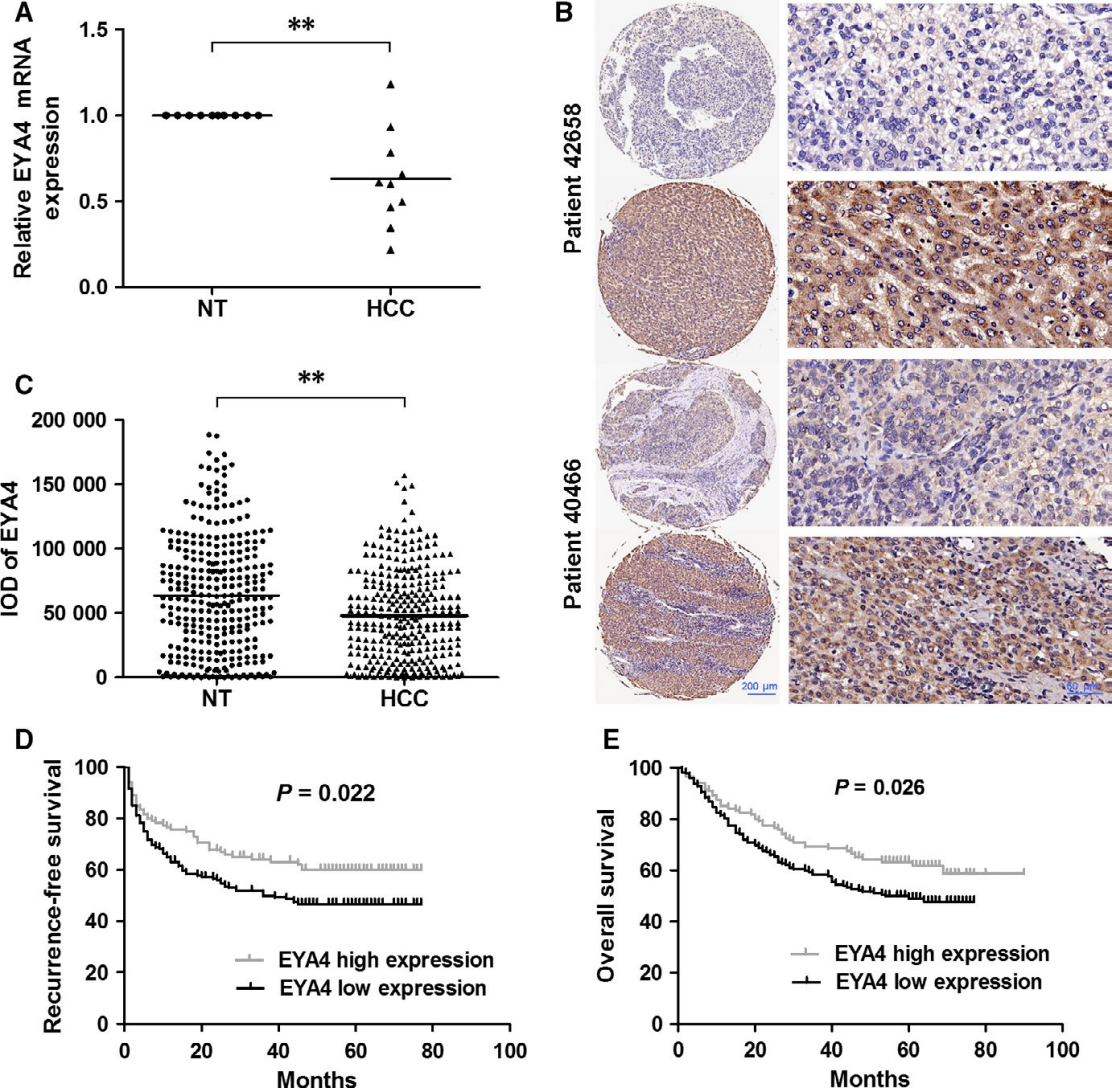
Eye absent homolog 4 (EYA4) is repressed in hepatocellular carcinoma (HCC) and serves as a prognostic factor among patients with HCC. (A) mRNA expression of EYA4 was determined by qRT‐PCR in HCC tissue samples and adjacent normal tissues. *β‐actin* was used as an internal control. (B) Representative immunohistochemical staining of EYA4 in the tissue microarray (TMA). (C) Normalized integrated optical density of EYA4 in HCC samples and paired non‐tumourous tissue samples in TMA analysis. The low‐EYA4‐expression group had worse recurrence‐free survival (D) and overall survival (E) as compared to the high‐EYA4‐expression group. The prognostic significance was evaluated by Kaplan‐Meier survival analysis and the log rank test. **P < 0.01

To analyse the expression of EYA4 in HCC, mRNA levels of EYA4 were detected in 10 HCC tissue samples and adjacent normal tissue samples by quantitative reverse‐transcription PCR (qRT‐PCR). Compared with the matched normal tissues, HCC tissue samples showed down‐regulation of *EYA4* mRNA (Figure 1A). We then performed IHC analysis on the TMA that contained 302 HCC samples to assess the clinical significance of EYA4. The expression was quantified in the Image‐Pro Plus 6.0 software (IPP). In agreement with the qRT‐PCR results, TMA analysis showed a significantly decreased intensity of EYA4 staining in HCC tissue samples than normal tissues (Figure 1B,C). Besides, EYA4 down‐regulation was significantly associated with tumour differentiation status but not other clinical parameters (Table 1). Furthermore, we sought to determine whether down‐regulation of EYA4 was associated with the prognosis of patients with HCC after hepatectomy. The log rank test revealed that patients with low EYA4 expression had unsatisfactory RFS and OS (Figure 1D,E). The univariate analysis indicated that HBs and HBe antigens, tumour size, tumour number, microvascular invasion and EYA4 expression remarkably correlated with RFS. Additionally, these parameters correlated significantly with OS (Table 2). Our multivariate analysis revealed that low EYA4 expression was an independent risk factor for RFS and OS among patients with HCC after curative hepatectomy (Table 3).

**Table 1:** Clinical characteristics of 302 hepatocellular carcinoma (HCC) patients according to Eye absent homolog 4 (EYA4) expression

Variable	EYA4 expression^a^		*P*‐value
	High	Low	
All cases	121	181	
Age (y), >60:≤60	28:93	35:146	0.471
Gender, male:female	105:16	159:22	0.860
HBe antigen positive:negative	26:95	37:144	0.855
HBs antigen positive:negative	101:20	161:20	0.171
Liver function, Child A:Child B	110:11	164:17	1.000
AFP (ug/L), >20:≤20	68:53	120:61	0.090
Tumour size (cm), >5:≤5	60:61	88:93	0.907
No. tumour, Solitary:Multiple	92:29	149:32	0.191
Differentiation, I + II:III + IV	18:103	13:168	0.035*
Micro‐vascular invasion, Present:Absent	72:49	109:72	0.905
Satellite nodules, Present:Absent	83:38	140:41	0.109
Recurrence, Present:Absent	64:57	113:68	0.121

Data are expressed as ratios.

a EYA4 downexpression was defined as integrated optical density in HCC weaker than paired non‐tumourous tissue.

* *P* < 0.05 by χ^2^ test.

**Table 2:** Univariate analysis of outcomes of 302 patients with hepatocellular carcinoma

Variable	Recurrence‐free survival(%)^a^	*P*‐value	Overall survival(%)^a^	*P*‐value
Age(year),>60:≤60	49.132:44.845	0.401	61.653:51.659	0.450
Gender, male:female	46.387:39.922	0.422	58.534:43.865	0.308
HBs antigen positive:negative	43.534:56.889	0.007*	55.607:58.641	0.012*
HBe antigen positive:negative	32.948:48.394	0.008*	41.431:59.976	0.020*
Liver function, Child A:child B	46.740:35.336	0.104	58.844:42.000	0.102
AFP(ug/L),>20:≤20	43.909:49.101	0.24	49.167:61.265	0.339
Tumour size(cm),>5:≤5	38.499:52.985	0.000*	43.950:65.939	0.000*
No. tumour, Solitary:multiple	48.203:35.930	0.009*	60.248:42.436	0.013*
Differentiation, I + II:III + IV	57.812:44.415	0.065	60.871:56.388	0.090
Micro‐vascular invasion, Present:absent	41.406:52.621	0.009*	52.848:59.325	0.012*
Satellite nodules, Present:absent	45.618:46.490	0.858	50.784:58.205	0.945
Eye absent homolog 4 expression High:low	51.554:42.007	0.022*	63.326:47.882	0.026*

a The time follow‐up ended is used to calculate the recurrence‐free survival and overall survival.

* *P* < 0.05 by long‐rank test.

**Table 3:** Multivariate analysis of several variables for overall survival and recurrence‐free survival

Variable^a^	Recurrence‐free survival		Overall survival	
	Hazard ratio (95% CI)	*P*‐value	Hazardratio(95% CI)	*P*‐value
HBs antigen, positive	0.485 (0.252‐0.931)	0.030*	0.518 (0.269‐0.995)	0.048*
HBe antigen, positive	0.658 (0.448‐0.967)	0.033*	0.725 (0.490‐1.0720	0.725
Tumour size(cm), >5	0.644 (0.450‐0.921)	0.016*	0.615 (0.430‐0.878)	0.008*
No. tumour, multiple	2.331 (1.530‐3.552)	0.000*	2.437 (1.600‐3.712)	0.000*
Differentiation, III + IV	0.871 (0.444‐1.709)	0.688	0.911 (0.466‐1.178)	0.785
Micro‐vascular invasion, present	0.732 (0.505‐1.061)	0.100	0.727 (0.500‐1.058)	0.096
Eye absent homolog 4 expression, low	1.541 (1.080‐2.199)	0.017*	1.540 (1.080‐2.197)	0.017*

a Variables were adopted for their prognostic significance by univariate analysis.

* *P* < 0.05 by Cox proportional hazards regression model.

### 3.2 EYA4 inhibits HCC cell migration, invasion and angiogenesis in vitro

**Figure 2:**
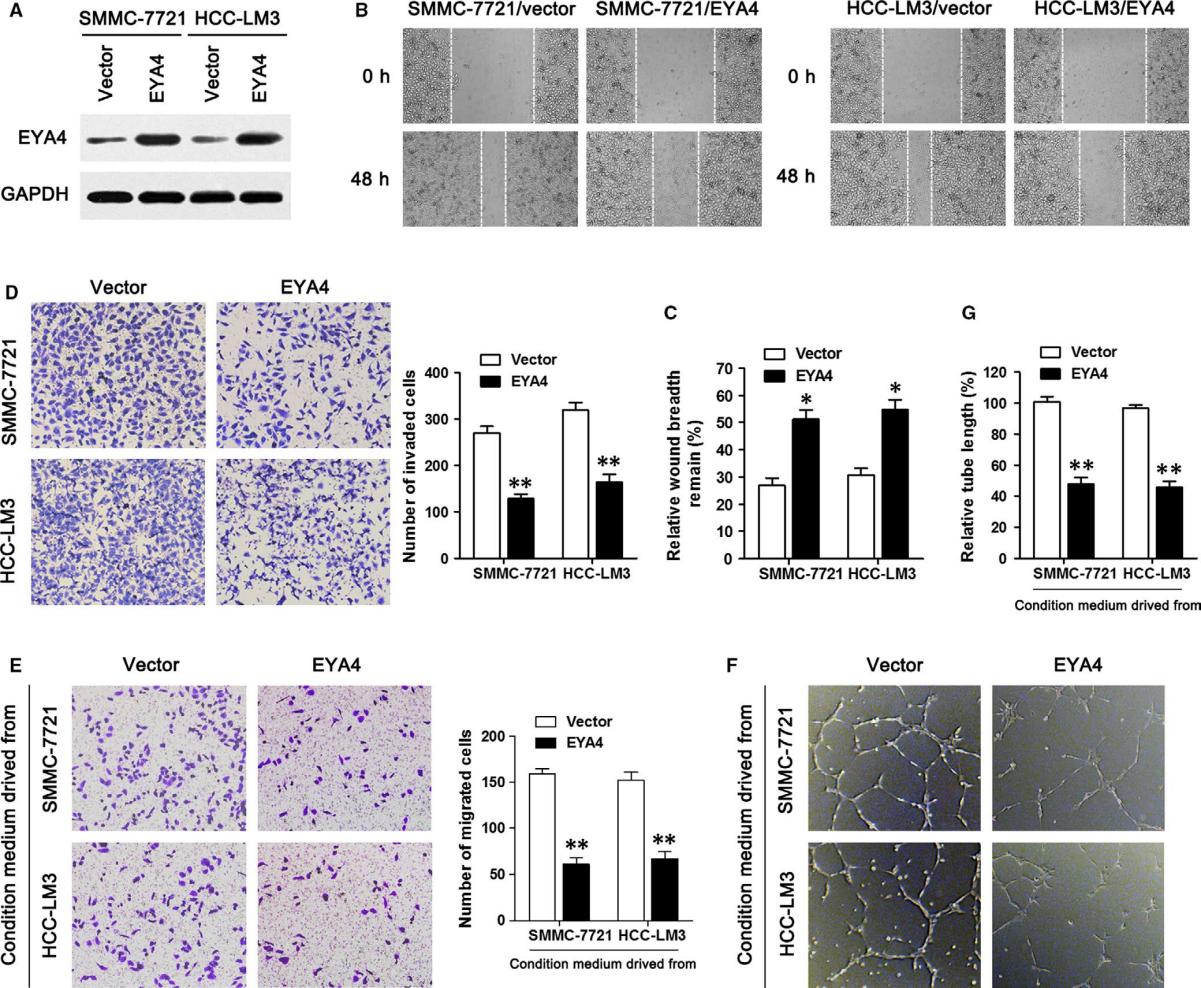
Eye absent homolog 4 (EYA4) inhibits hepatocellular carcinoma (HCC) cell migration and invasion, and attenuated HUVECs migration and tube formation in vitro. (A) Western blot detection of EYA4 protein expression in SMMC‐7721 and HCC‐LM3 cells transfected with the EYA4‐overexpressing lentiviral vector (indicated as “EYA4”) or empty vector (indicated as “Vector”). GAPDH was used as an internal control. (B) The wound healing assay was conducted to study the possible change in the cell migration ability after overexpression of EYA4 in SMMC‐7721 and HCC‐LM3 cells compared with their negative controls. Pictures were taken after scratching at the indicated time points. (C) The relative wound breadth remain (100%) represents the migration capacity of HCC cells, and the breadth at 0 h was set as 100%. (D) Transwell invasion assay was performed to investigate the cell invasion ability of HCC cells in which EYA4 was up‐regulated. The cells that passed through the membrane were counted. (E) Transwell migration assay of HUVECs cultured with EYA4‐overexpressing HCC cell medium. (F) Representative pictures of tube formation were taken at 8 h post‐plating and quantified for tubule length (G). The data are presented as the mean ± SD. All the experiments were conducted three times. *P < 0.05, **P < 0.01

To evaluate the possible function of EYA4 in HCC cells, we introduced the EYA4‐overexpressing lentivirus into SMMC‐7721 and HCC‐LM3 cells. The efficiency of the lentiviral transduction was verified by Western blotting (Figure 2A). Wound‐healing and Transwell invasion assays were first conducted to explore the function of EYA4 in HCC cells. As shown in Figure 2B,C, 48 h after administration of a scratch, the gap between cells was broader in SMMC‐7721 and HCC‐LM3 cell groups overexpressing EYA4 than that in the negative control group, indicating that overexpression of EYA4 could reduce cell migration. In additon, the results of the Transwell invasion assay revealed that ectopic expression of EYA4 weakened the invasion ability of HCC cells (Figure 2D). Subsequently, we examined the effects of EYA4 overexpression on the angiogenesis using an in vitro HUVEC model. The conditioned media from EYA4‐overexpressing SMMC‐7721 and HCC‐LM3 cells significantly attenuated the migration and tube‐like structure formation of HUVECs (Figure 2E‐G), suggesting that EYA4 inhibits HCC angiogenesis in vitro.

### 3.3 EYA4 regulates HCC cell invasion and angiogenesis through the c‐JUN/VEGFA pathway

**Figure 3:**
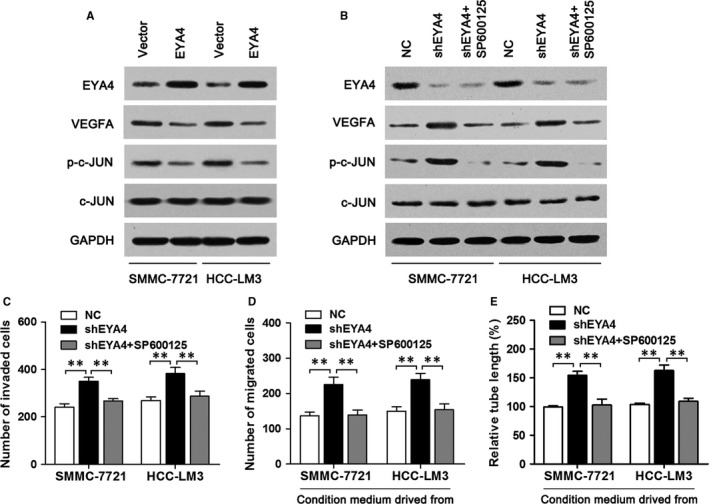
Eye absent homolog 4 (EYA4) suppresses angiogenesis via down‐regulating c‐JUN/VEGFA pathway. (A) Western blotting was performed to examine the protein levels of EYA4, VEGFA and p‐c‐JUN in SMMC‐7721 and hepatocellular carcinoma (HCC)‐LM3 cells transfected with the EYA4‐overexpressing lentiviral vector (indicated as “EYA4”) or empty vector (indicated as “Vector”). GAPDH served as the loading control. (B) Western blot analyses of EYA4, VEGFA and p‐c‐JUN in EYA4 knockdown SMMC‐7721 and HCC‐LM3 cells with or without SP00125 treatment (20 μmol/L). (C) The cell invasiveness was determined by Transwell invasion assay in the indicated HCC cells. (D) Transwell migration assay of HUVECs cultured in the conditions of the indicated HCC cells. (E) Matrigel capillary tube formation assay of HUVECs under the treatment of the indicated cell culture medium. Values are presented as the mean ± SD. **P < 0.01

Next, we studied the underlying mechanism via which EYA4 suppresses HCC cell invasiveness and angiogenesis. Given that VEGFA was crucial for cancer growth and neovascularization,^15^ we examined the expression of VEGFA in SMMC‐7721 and HCC‐LM3 cells overexpressing EYA4 or vector. We observed that overexpression of EYA4 decreased the levels of VEGFA in these cells (Figure 3A). Previous studies have shown that VEGFA could be regulated by c‐JUN and the expression of c‐JUN was down‐regulated in HCT15 colorectal cancer cells overexpressing EYA4.^10^, ^15^ Of note, EYA4 possessed the N‐terminal serine/threonine‐specific phosphatase activity.^7^ So we postulated that EYA4 regulates HCC cell invasion and angiogenesis through targeting c‐JUN/VEGFA. To test this hypothesis, we measured the total and phosphorylated (p) c‐JUN expression in EYA4 overexpressing cells or control cells and the result showed that EYA4 overexpression reduced the phosphorylation of c‐JUN (Figure 3B). On the contrary, EYA4 knockdown increased the levels of p‐c‐JUN and VEGFA and this increase was attenuated by treatment with SP600125 (an inhibitor of the JNK/c‐JUN pathway; Figure 3B). Furthermore, inhibition of c‐JUN can block EYA4 knockdown‐mediated promotion of HCC cell invasion (Figure 3C). We also found that after c‐JUN inhibition, conditioned media from EYA4 knockdown‐transfected HCC cell cultures could significantly decrease the migration and capillary tube formation of HUVECs (Figure 3D,E).

### 3.4 EYA4 attenuates tumour angiogenesis and metastasis in vivo

**Figure 4:**
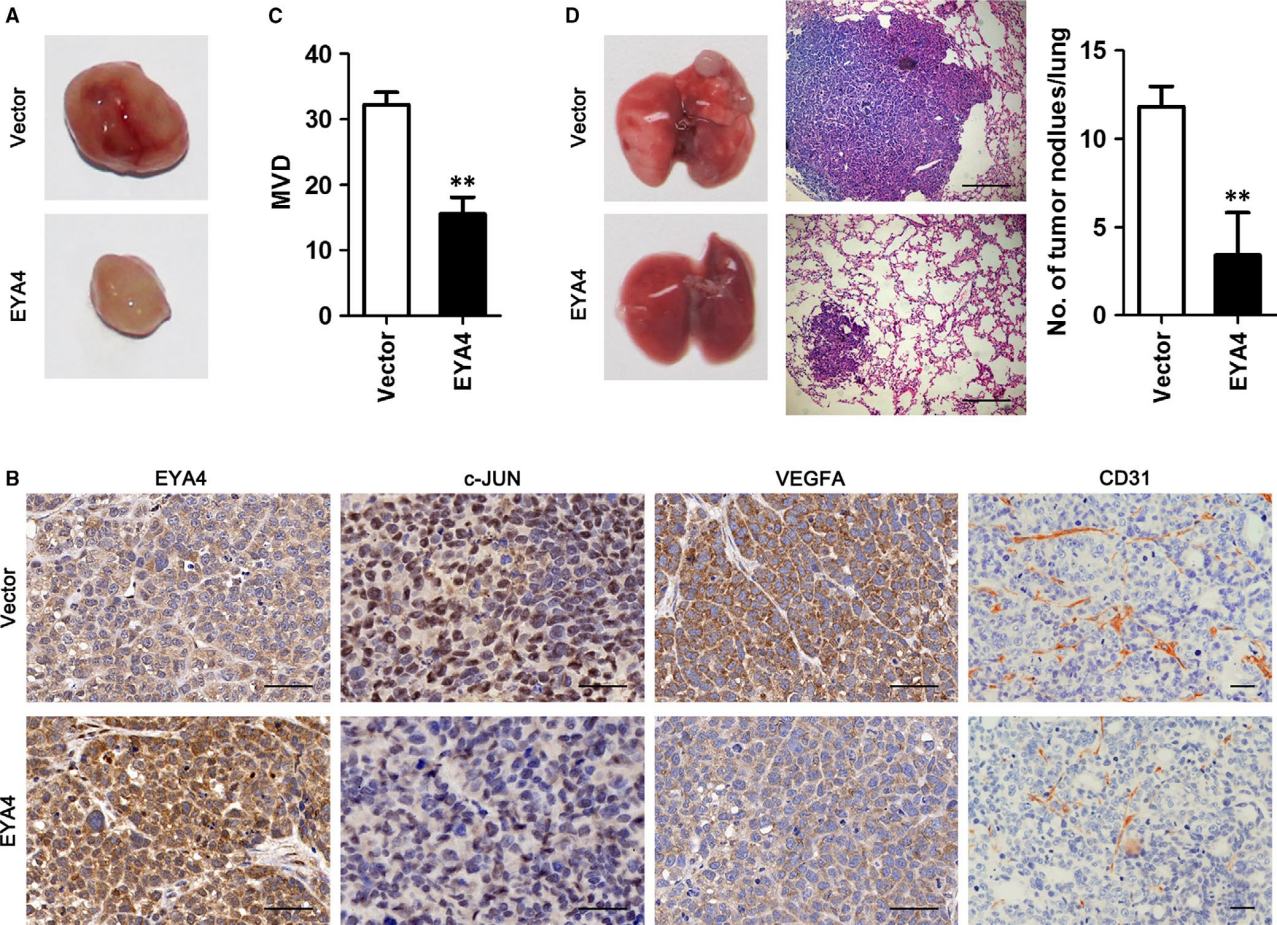
Eye absent homolog 4 (EYA4) attenuates tumour angiogenesis and metastasis in vivo. (A) Morphological observation of tumours formed after 4 weeks. (B) Immunohistochemical staining of EYA4, c‐JUN, VEGFA and CD31 was performed on serial sections of tumours from hepatocellular carcinoma (HCC)‐LM3/vector group and HCC‐LM3/EYA4 group. Scale bar = 50 μm. (C) Histograms showed microvessel density (MVD) in each group. (D) EYA4 suppresses tumour metastasis in vivo. (Left) Representative bright‐field imaging of the lungs; (middle) haematoxylin and eosin (H&E) staining was performed on serial sections of metastatic tumours (right). The number of lung metastatic lesions was determined. Scale bar = 200 μm.**P < 0.01

Finally, we determined whether EYA4 overexpression could regulate HCC angiogenesis and metastasis in vivo. To this end, HCC‐LM3 cells stably expressing EYA4 or empty vector were injected subcutaneously into nude mice. Palpable tumours formed 1 week after implantation. The mice were sacrificed and the tumours were recovered after four weeks (Figure 4A). As shown in Figure 4B, EYA4 overexpression suppressed the expression levels of c‐JUN and VEGFA in HCC tumours. Additionally, EYA4 overexpression led to a significant decline in the CD31‐positive MVD (Figure 4B,C). To further determine the effect of EYA4 on in vivo tumour metastasis, HCC‐LM3 stably expressing EYA4 or empty vector were injected into nude mice through the tail vein. Eight weeks later, the lungs were prepared for standard histological examination (Figure 4D). The number of tumour foci found in the lungs in the HCC‐LM3/vector group was much higher than in the HCC‐LM3/EYA4 group (Figure 4D). These results indicated that EYA4 strongly suppresses HCC tumour angiogenesis and metastasis in vivo.

## 4 DISCUSSION

In this study, we revealed that EYA4 was significantly down‐regulated in clinical HCC samples and the down‐regulation of EYA4 was associated with poor RFS and OS among patients with HCC. Overexpression of EYA4 suppressed HCC cell migration, invasion and capillary tube formation of endothelial cells in vitro, as well as in vivo tumour angiogenesis and metastasis in a mouse model. Mechanistically, EYA4 could inhibit HCC angiogenesis and metastasis by inhibiting c‐JUN/VEGFA pathway. Our results suggest that the down‐regulation of EYA4 promotes HCC progression, and EYA4 may be a novel molecular prognostic marker of HCC.

EYA4 was first identified by Borsani *et al* in 1999 and has been reported to be dysregulated in a variety of human cancers.^16^ Hypermethylation and underexpression of EYA4 have been detected in both major non–small cell lung cancer subtypes and at the earliest stages of lung cancer.^9^ EYA4 expression is significantly reduced in pancreatic ductal adenocarcinoma (PDAC) tissues and PDAC patients with down‐regulated EYA4 expression in the tumour have shorter OS and DFS.^17^ The *EYA4* gene has been found to be hypermethylated in HCC, and suppression of the *EYA4* gene is associated with worse DFS and OS in 45 patients with HCC.^11^ Consistent with these findings, this study involved more clinical samples and showed that EYA4 is repressed in HCC tissue samples compared with adjacent healthy tissues from 302 patients with HCC. Of note, the log rank test showed that patients with low EYA4 expression exhibited unsatisfactory RFS and OS. A multivariate analysis revealed that low expression of EYA4 is an independent prognostic indicator of RFS and OS among patients with HCC. These results confirmed that EYA4 may be a prognostic biomarker of HCC.

Most studies suggest that EYA4 functions as a tumour suppressor by affecting cell proliferation and growth, migration and invasiveness of some human cancers, such as colorectal cancer, PDAC and intrahepatic cholangiocarcinoma.^18^ Similarly, our functional study suggests that overexpression of EYA4 restrains HCC cell migration and invasion. What is more, ectopic expression of EYA4 inhibited the metastatic activity of HCC cells in nude mice. Notably, angiogenesis, one of the pivotal cancer hallmarks, is essential for tumorigenesis, progression and prognosis.^19^ We found that EYA4 overexpression interfered with the pro‐angiogenic activity of HCC cells, as determined by in vitro endothelial cell tube formation assay. Additionally, VEGFA expression was suppressed by EYA4, which provides an explanation for the decreased angiogenic activity in HCC cells. Hence, our data support the tumour suppressor function of EYA4 in HCC.

Although EYA4 was found to be involved in the regulation of tumour progression in HCC, the underlying molecular mechanism remains poorly understood. Previously Kim and colleagues reported that EYA4 acts as a new tumour suppressor gene in colorectal cancer and its expression was down‐regulated in HCT15 colorectal cancer cells overexpressing EYA4.^12^ As a transcription factor, c‐JUN functions as an upstream regulator of many genes, including VEGFA and participates in tumour growth and metastasis.^20^, ^21^ Since EYA4 possessed the N‐terminal serine/threonine‐specific phosphatase activity,^7^ it was hypothesized that EYA4 down‐regulated c‐JUN via dephosphorylation. In this study, we found that EYA4 overexpression reduced the phosphorylation of c‐JUN. On the contrary, EYA4 knockdown increased the levels of p‐c‐JUN and VEGFA, and this increase was attenuated after c‐JUN inhibition. Furthermore, c‐JUN inhibition largely attenuated the positive effects of the EYA4 knockdown on the angiogenesis and invasiveness of HCC cells, indicating that EYA4 regulates HCC angiogenesis and metastasis through the c‐JUN/VEGFA pathway.

In conclusion, our results illustrate that down‐regulation of EYA4 may be a promising predictor of HCC progression. Down‐regulation of EYA4 strongly facilitates HCC cell migration, invasiveness and angiogenesis and this phenomenon is mainly mediated by the c‐JUN/VEGFA pathway. Suppression of tumour progression through EYA4 overexpression holds promise as a method for HCC treatment.

## CONFLICT OF INTEREST

None.

## AUTHOR CONTRIBUTIONS

FMG and WPZ were involved in the study conception and design. FMG, SXY, LL and WPZ collected and analysed the data. WPZ, YY and ZYP provided materials. FMG wrote the paper.
